# The FBP1‐TP53‐NRF2 metabolic switch in metabolic dysfunction‐associated steatohepatitis‐hepatocellular carcinoma progression and senescence reversal

**DOI:** 10.1002/ctm2.70293

**Published:** 2025-03-30

**Authors:** Yahui Zhu, Donglin Wei, Michael Karin, Li Gu

**Affiliations:** ^1^ School of Medicine Chongqing University Chongqing China; ^2^ Departments of Pharmacology and Pathology Laboratory of Gene Regulation and Signal Transduction School of Medicine, University of California San Diego (UCSD) La Jolla California USA; ^3^ Department of Laboratory Medicine West China Hospital, Sichuan University Chengdu China; ^4^ Clinical Laboratory Medicine Research Center West China Hospital, Sichuan University Chengdu China

Metabolic dysfunction‐associated steatohepatitis (MASH) is defined by extensive hepatosteatosis, liver injury, persistent inflammation and fibrosis.^1^ Around 2% of patients with MASH progress to MASH‐related hepatocellular carcinoma (MASH‐HCC) every year, indicating the importance of MASH as the newly emerging HCC aetiology.^2^ Nonetheless, it is not understood how MASH progresses to HCC and how MASH is maintained in some patients without progression to HCC. Here, we outline our recently discovered mechanism by which energy‐dense diets induce hepatocyte senescence, previously presumed to prevent HCC progression. Using fructose and fat‐rich diets we found diet‐induced hepatocyte single‐strand DNA breaks that lead to activation of the DNA damage response (DDR), which culminates in the activation of TP53 and induction of its targets p21^CIP1^ and p16^INK4a^, two cell‐cycle inhibitors that enter DNA damaged hepatocytes into senescence, a state during which they cannot proliferate. Interestingly, we found that TP53 also leads to the induction of the metabolic enzyme FBP1, which we previously identified as an AKT inhibitor due to its ability to interact with both AKT and PP2A catalytic subunit, which inactivates AKT.^3^ By inhibiting AKT, FBP1 which also acts as an HCC‐specific tumour suppressor,^4^ leads to stabilization of TP53, thereby boosting hepatocyte senescence^5^ (Figure 1). FBP1 is upregulated during MASH, but as MASH progresses to HCC FBP1 is degraded and leads to activation of AKT, in insulin and growth factor‐stimulated hepatocytes. By phosphorylating MDM2 and enhancing its ability to induce TP53 degradation,^6^ the downregulation of FBP1 also leads to TP53 deficiency, thereby allowing senescent hepatocytes to re‐enter the cell cycle. Conversely, the upregulation of FBP1 in response to energy‐dense and DNA‐damaging diets inhibits the phosphorylation of GSK3α/β, thereby increasing the substrate binding activity of these kinases which phosphorylate NRF2 and β‐catenin and thereby triggering their degradation, resulting in low NRF2 expression in senescent hepatocytes.^5^ However, sustained metabolic stress and autophagy disruption lead to accumulation of p62/SQSTM1 which sequesters the major negative regulator of NRF2, KEAP1. This results in NRF2 activation, which induces the expression of ERK1/2‐activating EGF and PDGF family members and phosphorylation‐directed and TRIM28‐dependent FBP1 degradation (Figure 1). FBP1 degradation, as discussed above, also results in TP53 degradation.

**FIGURE 1:**
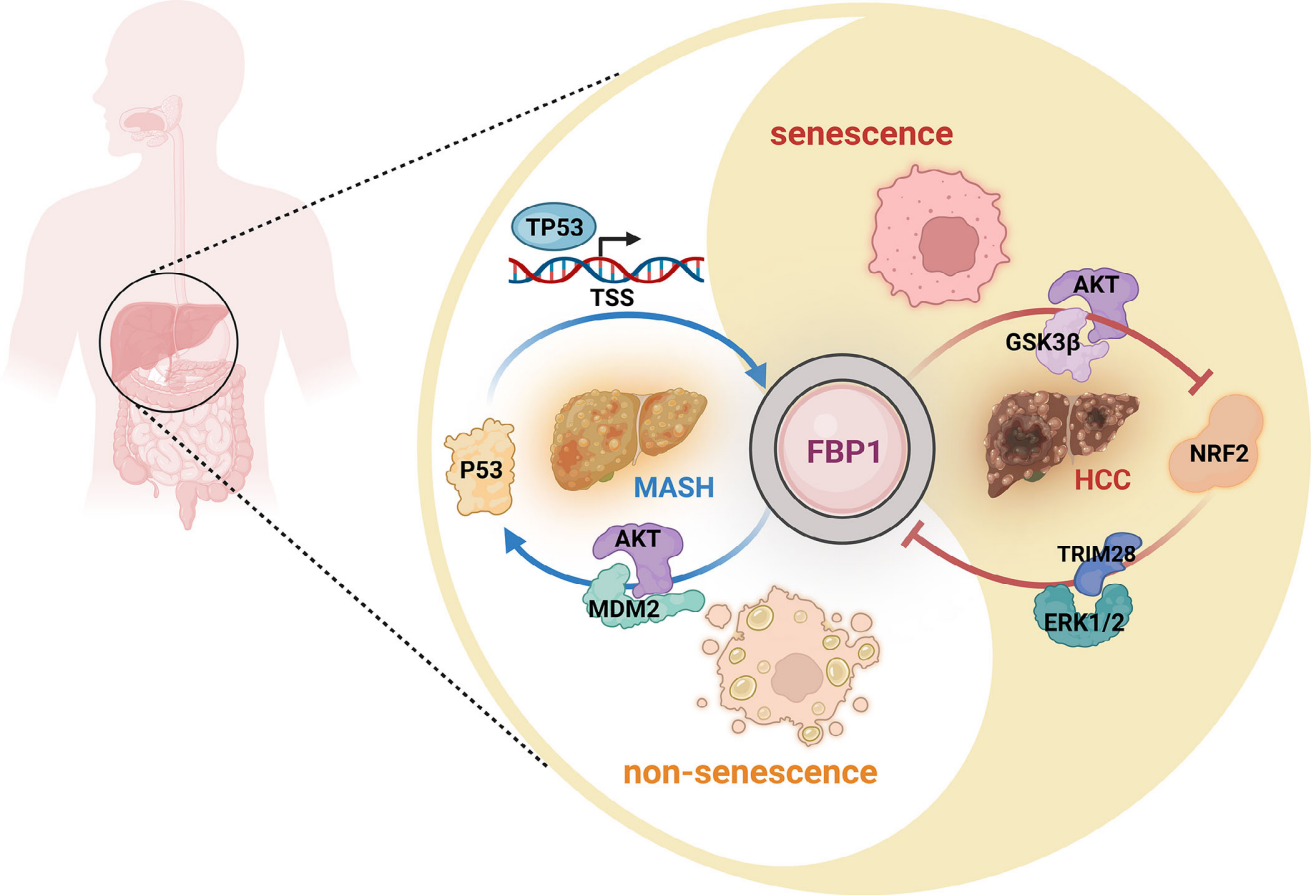
The Yin and Yang of FBP1‐P53 and NRF2‐FBP1 in metabolic dysfunction‐associated steatohepatitis (MASH) and MASH‐related hepatocellular carcinoma (MASH‐HCC). Senescence controlling the FBP1‐NRF2‐P53 auto‐regulatory loop initiates cancer in metabolically stressed human and mouse livers.

On the one hand, after oncogene activation, senescence‐induced immune surveillance by CD8^+^ T cells clears premalignant cells and inhibits liver tumorigenesis.^7^ However, MASH is associated with liver fibrosis driven by transforming growth factor‐β, which also promotes the immunoglobulin M (IgM) to IgA class‐switch which leads to the appearance of immunosuppressive plasma cells that inhibit hepatic immunosurveillance.^8^ This promotes the accumulation of senescent hepatocytes, a common feature of human MASH.^9^, ^10^ As discussed above, some of the senescent hepatocytes find a way to bypass the senescent state and immune surveillance and rapidly progress to HCC.^5^ Although the accumulation of senescent cells during old age was proposed to enhance tumour‐promoting smouldering inflammation through the senescence‐associated secretory phenotype,^11^ the FBP1‐NRF2 crossregulatory interactions described above seem to play a more critical role in MASH to HCC progression. Moreover, through lineage tracking studies we were able to demonstrate that malignant HCC cells directly descend from senescent hepatocytes.^5^ Other studies have proposed that senescent cells which resumed proliferation enter a new state different from cells that never senesced.^12^ Thus, finding ways to specifically target senescent cells that have re‐entered the cell cycle could lead to novel HCC preventives and therapeutics.

## 1 CLINICAL IMPLICATION AND FUTURE DIRECTIONS

More than 10 years ago, He et al. made an important advance by identifying HCC progenitor cells (HcPC) which are present in collagenase‐resistant hepatocyte aggregates from livers of carcinogen (DEN) treated mice that resemble hepatobiliary stem cells.^13^ More recently, Carlessi et al., working in Australia, have used single nucleus (snRNA‐seq) technology to identify the disease‐associated hepatocytes (DaHep) in fibrotic/cirrhotic livers that can predict HCC progression.^14^ By collaborating with Drs Carlessi and Tirnitz‐Parker we found that the FBP1‐TP53‐NRF2 axis may also account for the appearance of both HcPC and daHep and their progression to frank HCC.^5^ Thus, alterations in FBP1 and NRF2 expression may have a predictive value. Indeed, the downregulation of FBP1 expression in human HCC correlated with promoter hypermethylation of the *FBP1* gene.^5^ It remains to be seen whether the *FBP1* promoter region first undergoes hypermethylation in HcPC/daHep at a point that precedes the appearance of histologically or radiologically detected HCC.

## CONFLICT OF INTEREST STATEMENT

The authors declare no conflict of interest.

## ETHICS STATEMENT

Not applicable.

## Data Availability

Data sharing is not applicable to this article as no datasets were generated or analyzed during the current study.
